# Trace Analysis by Degrees: An Academic Perspective

**DOI:** 10.6028/jres.093.014

**Published:** 1988-06-01

**Authors:** George H. Morrison

**Affiliations:** Baker Laboratory of Chemistry, Cornell University, Ithaca, NY 14853

The increasing awareness of the important role of very small amounts of chemical species in chemical, physical, and biological systems has greatly stimulated the refinement and extension of analyses at these levels. The analytical requirements imposed by the minute quantities and typically complex systems involved has led to the development of methodology and instrumentation so specialized as to warrant consideration as a distinct field of analytical chemistry—trace analysis. One of the aims of this symposium is to gain an accurate assessment of the current state of this field, and this paper is an attempt to present this status from an academic perspective.

In order to discuss any specialty in analytical chemistry as carried on in colleges and universities, it is necessary to first review the current status of the whole discipline. It must be stated that the main missions of universities are teaching and research, and chemistry, one of the basic sciences, is an essential discipline in most colleges and universities. How does analytical chemistry fare in academe? A review of the American Chemical Society Directory of Graduate Research lists faculties, publications and theses in chemistry and other related disciplines at universities in the United States and Canada. A review of the 1985 edition, the latest available, reveals that of the 315 chemistry departments listed, 201 include analytical chemistry with 510 analytical faculty members. It is obvious that these departments cover a wide range of sizes, so that some of the smaller institutions are less structured. Of these 201 departments, 80 have at least three or more analytical chemistry faculty members. What becomes quite apparent from a review of the data is that while analytical chemistry is not formally included in every department, there are a significant number of strong analytical departments in the United States and Canada to advance the science adequately. In this environment we can now turn our attention to trace analysis.

With regard to trace analysis research, two major approaches are evident: development of new techniques and methodology, and application to important problems. Because the field of trace analysis is so broad, I have chosen to limit my evaluation to trace element analysis, the area of my expertise. This specialty can be conveniently divided into three categories: bulk analysis, spatially resolved analysis, and speciation. In the area of applications, trace analytical research is being pursued by a good number of faculty to solve important biomedical, environmental, solid state materials, and geochemical problems.

Among the techniques currently being employed for bulk trace analysis, electroanalytical, atomic spectroscopy, x-ray fluorescence, activation analysis, and mass spectrometry are the most advanced and most used. A review of bulk trace techniques over the past 40 years indicates continuous progress in each of these categories. See figure 1. Thus, each of these techniques has continuously been used and kept viable with novel research advances. The current exciting areas in each of these categories include modified electrodes, inductively coupled plasma spectroscopy (ICP), proton induced x-ray emission spectroscopy (PIXE), charged particle and prompt gamma activation analyses, and ICP-mass spectroscopy.

While there will always be a need for more sensitive bulk trace methods of broad scope, there has been increased interest in recent years in localization of elements in solid samples. These spatially resolved methods of trace analysis can establish the distribution of many elements with spatial resolutions at the micrometer level or better. Table 1 lists some of the more popular methods, their resolution and sensitivity. Included are electron probe microanalysis (EPMA), scanning Auger microscopy (SAM), secondary ion mass spectrometry (SIMS), laser microprobe mass analysis (LAMMA) and proton induced x-ray emission spectroscopy (PIXE). The object of these spatially resolved methods is to correlate elemental concentration with morphology to explain the properties of various heterogeneous samples in solid state, metallurgical, biological, geological, etc. problems.

One of the more powerful spatially resolved methods, SIMS or ion microscopy, can determine the distribution of all elements at the ppm level or better. Both stigmatic and scanning ion microscopes are being used with spatial resolutions of 50–100 nm. Of particular importance is the use of the technique for subcellular elemental mapping of tissue sections and cultured cells to solve physiological, pathological, and toxicological problems in biology and medicine.

The third category in trace element analysis is the study of the speciation of an element, i.e., the determination of the various individual physicochemical forms. This is of great interest, especially in environmental analysis, because the toxicity of an element depends on its chemical form. Similarly in biology and medicine, elements such as chromium can be associated with several diseases while in the Cr(VI) state, however, Cr(III) is relatively non-toxic. Thus, elements can be present in many forms in samples, including the zerovalent metal, the free ion, an organometallic complex or a chelate. These different chemical forms of an element greatly influence its toxicity, bioavailability, bioaccumulation, and transport. Unfortunately, very little has been done to date in the area of speciation analysis. What has been done involves the use of a variety of separation and concentration techniques followed by the use of one of a number of detection methods. As can be appreciated, the use of these wet chemical methods requires great care to ensure the preservation of the chemical form of the species during the analysis. Some physical techniques can provide information on the chemical state of elemental species in solids. They include x-ray photoelectron spectroscopy (XPS), Auger electron spectroscopy (AES including SAM), and x-ray absorption fine structure spectroscopy (EXAFS). Current trends in trace element analysis include preconcentration methodology, multi-element methods, hybrid methods, and single atom detection.

Thus, the field of trace element analysis has continued to grow vigorously over the years with the advent of new and more sophisticated instrumentation and the increasingly more demanding needs for complete characterization of complex systems in science and technology. The colleges and universities have contributed significantly to this advancement through both fundamental research and in educating generations of students who have continued to work in this exciting field in university, industrial, and government laboratories.

## Figures and Tables

**Figure 1:**
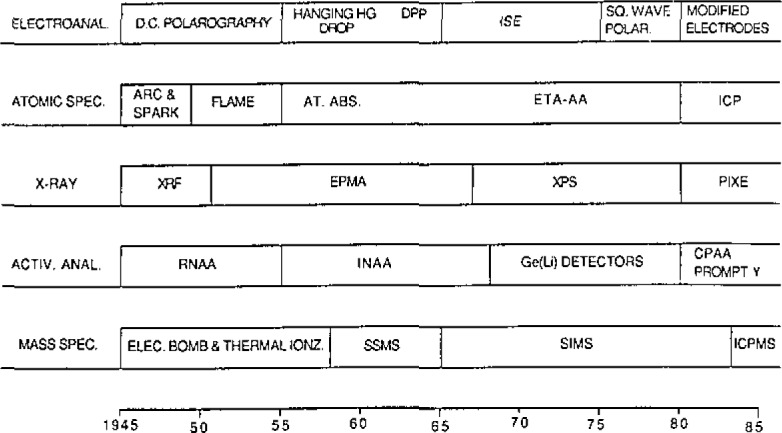
Progress in trace element analysis.

**Table 1:** Spatially resolved methods in trace element analysis

Method	Resolution	Sensitivity^a^
EPMA	0.01 – 1 *μ*m	ppth
SAM	0.05 – 0.5 *μ*m	ppth
SIMS	0.1 – 0.5 *μ*m	ppth–ppm
LAMMA	0.5 – 1 *μ*m	ppm
PIXE	500 – 2500 *μ*m	ppm–ppb

a ppth—parts per thousand; ppm—parts per million; ppb—parts per billion.

